# Through thick and thin: Risk of venous thromboembolism vs bleeding after abdominal transplant

**DOI:** 10.1016/j.sopen.2023.06.006

**Published:** 2023-06-21

**Authors:** Jenna N. Whitrock, Adam D. Price, Shimul A. Shah

**Affiliations:** Department of Surgery, University of Cincinnati College of Medicine, United States of America

Venous thromboembolism (VTE) is one of the dreaded complications that can occur after abdominal surgery and may lead to increased length of hospitalization, risk of future VTE, and risk of death [1]. The manuscript entitled “Risk and Predictors Associated with Venous Thromboembolism Following Abdominal Transplantation” outlines the importance of VTE after abdominal organ transplant (liver, kidney, pancreas). In their analysis of the Nationwide Readmissions Database (NRD), the authors noted a 1.96 % rate of VTE after abdominal transplant with individual rates for pancreas, liver, and kidney transplants at 5.6 %, 3.4 %, and 1.6 %, respectively. Their analysis revealed an overall increased risk of in-hospital mortality (AOR 3.95, 95%CI 2.93–5.34) for those with VTE as well as increased risk of cardiac complications (AOR 2.12, 95%CI 1.65–2.72), infectious complications (AOR 3.66, 95%CI 2.92–4.58), and respiratory complications (AOR 2.4, 95%CI 1.98–2.92), all highlighting the significance of the VTE after transplant. In addition to medical morbidity, the authors also noted an average increase in cost per patient after VTE of around $60,000, adding financial burden to the healthcare system and payors. Patients were also less likely to be discharged to home and were more likely to be readmitted at 90 days, thus suggesting a likely significant under-estimation of the total cost-burden of VTE.

In their analysis, the authors noted that those with increased comorbidities, defined by the Elixhauser comorbidity index, were more likely to experience VTE. Other markers for increased patient complexity that were noted to be associated with the VTE group included recent weight loss, electrolyte abnormalities, history of diabetes mellitus, peripheral vascular disease, congestive heart failure, and cardiac arrhythmias during admission for transplant. Those with a history of VTE or hypercoagulability disorders were also more likely to experience VTE. However, they also noted that for pancreas transplant, the VTE and non-VTE groups were very similar, potentially suggesting that procedural factors may be more important in this group.

Despite the known risks of VTE after abdominal transplant, the risk of bleeding must also be addressed. Though controversy on the subject exists, the concern for bleeding-related complications frequently plays a role in decision making regarding the timing and use of VTE prophylaxis [2]. Bleeding after abdominal transplant can lead to an increase in mortality as well as graft loss, increased hospital stay, cost, and need for re-operation [3,4]. When there is increased concern for bleeding after abdominal transplant, VTE prophylaxis is more frequently held. Though the authors were unable to evaluate the rate of bleeding complications in this dataset, further work should be conducted to evaluate this phenomenon in relation to timing and use of VTE prophylaxis and rates of VTE. We recommend VTE prophylaxis in all patient immediately after transplant. A specific population where this is commonly missed is the ill end-stage liver disease patient admitted due to concerns for bleeding but in fact these patients may be hypercoaguable and need VTE prophylaxis.

This study does have some noteworthy limitations. First, this study only included those who received an abdominal transplant within 48 h of admission to the hospital. This could potentially lead to bias in the study as it excludes the sickest individuals who may have been hospitalized prior to 48 h before transplant. Therefore, the applicability of study findings may be limited to those who were not sick enough to require long-term hospitalization preoperatively. Other limitations which were discussed by the authors included lack of granular data regarding donor characteristics, operative details, prior anticoagulation use, hospital-based VTE prophylaxis protocols, and lab values such as thromboelastography. Finally, the only cases of VTE captured included those diagnosed on admission or readmission, therefore excluding any VTE diagnosed and treated as an outpatient.

As outlined by the authors, VTE after abdominal transplant is a serious issue that leads to significant morbidity, mortality, and cost burden. VTE protocols are not standardized across transplant programs and are often institution based and dependent on individual clinician judgement. Bleeding risk is likely one of the major factors that influences decision-making regarding timing of VTE prophylaxis in patients after transplant, though further work needs to be completed to really understand the risk of bleeding versus VTE. The use of VTE prophylaxis after discharge is also controversial, and further work in this realm is also needed. Having a better understanding of these conflicting problems could help lead to creation of standardized protocols that could help decrease the burden of VTE and bleeding after transplant, ultimately leading to better outcomes for patients.

All authors contributed equally to the manuscript in preparation and revisions. There is no funding source. And ethical approval is n/a. No IRB necessary for invited commentary.

## Declaration of competing interest

There are no conflicts of interest.
